# Failing to Plan Is Planning to Fail: Utilization of Advance Care Directives in Older Adults Living With HIV

**DOI:** 10.1093/geroni/igab046.845

**Published:** 2021-12-17

**Authors:** Paul Nash, Annie Nguyen, Anna Egbert, Mark Brennan-Ing, Stephen Karpiak

**Affiliations:** 1 University of Southern California, Los Angeles, California, United States; 2 University of Southern California, Alhambra, California, United States; 3 Ronin Institute, Montclair, New Jersey, United States; 4 Brookdale Center for Healthy Aging Hunter College, CUNY, New York, New York, United States; 5 GMHC, National Resource Center on Aging and HIV, New York, New York, United States

## Abstract

Advance Care Planning (ACP) makes up an integral part of the care continuum, especially for those living with chronic conditions such as HIV. Little research exists to understand how intersections of race, gender, sexuality and gender identity combine to influence the choices made by older adults living with HIV regarding ACP. The Research on Older Adults with HIV (ROAH) 2 study collected data from across the US and investigated the incidence and range of ACP amongst those 50+ living with HIV. Correlational analysis indicated that being White was significantly related to having at least one directive (R=0.070, p=0.035) where being African American correlated negatively with several forms of ACP. Additionally, there were also significant relationships between being Transgender, being gay, and being a woman as to the engagement with ACP options. Further analysis explored the impact of finance, self-rated health and social support networks.

